# Physical Activity Counselling during Pulmonary Rehabilitation in Patients with COPD: A Randomised Controlled Trial

**DOI:** 10.1371/journal.pone.0144989

**Published:** 2015-12-23

**Authors:** Chris Burtin, Daniel Langer, Hans van Remoortel, Heleen Demeyer, Rik Gosselink, Marc Decramer, Fabienne Dobbels, Wim Janssens, Thierry Troosters

**Affiliations:** 1 KU Leuven, Faculty of Kinesiology and Rehabilitation Sciences, Leuven, Belgium; 2 KU Leuven, Respiratory Rehabilitation and Respiratory Division, University Hospitals, Leuven, Belgium; 3 Rehabilitation Research Centre, Biomedical Research Institute, Faculty of Medicine and Life Sciences, Hasselt University, Diepenbeek, Belgium; 4 Department of Public Health and Primary Care, KU Leuven, Leuven, Belgium; Weill Cornell Medical College in Qatar, QATAR

## Abstract

**Background:**

Pulmonary rehabilitation programs only modestly enhance daily physical activity levels in patients with chronic obstructive pulmonary disease (COPD). This randomised controlled trial investigates the additional effect of an individual activity counselling program during pulmonary rehabilitation on physical activity levels in patients with moderate to very severe COPD.

**Methods:**

Eighty patients (66±7 years, 81% male, forced expiratory volume in 1 second 45±16% of predicted) referred for a six‐month multidisciplinary pulmonary rehabilitation program were randomised. The intervention group was offered an additional eight-session activity counselling program. The primary outcomes were daily walking time and time spent in at least moderate intense activities.

**Results:**

Baseline daily walking time was similar in the intervention and control group (median 33 [interquartile range 16–47] vs 29 [17–44]) whereas daily time spent in at least moderate intensity was somewhat higher in the intervention group (17[4–50] vs 12[2–26] min). No significant intervention*time interaction effects were observed in daily physical activity levels. In the whole group, daily walking time and time spent in at least moderate intense activities did not significantly change over time.

**Conclusions:**

The present study identified no additional effect of eight individual activity counselling sessions during pulmonary rehabilitation to enhance physical activity levels in patients with COPD.

**Trial Registration:**

clinicaltrials.gov NCT00948623

## Introduction

Patients with Chronic Obstructive Pulmonary Disease (COPD) are markedly inactive in daily life [1]. Physical inactivity plays a crucial role in the development of systemic consequences of COPD including skeletal muscle weakness and osteoporosis [2]. Furthermore, low physical activity levels have been associated with decreased exercise capacity [3], increased hospital admission rate and increased mortality [4]. Based on these observations, daily physical activity behaviour should be a key target for therapeutic interventions aiming to induce long-term health benefits.

Even though pulmonary rehabilitation leads to clinically important improvements in terms of exercise tolerance [5, 6] and whole-body endurance capacity almost doubles [7], the observed benefits do not consistently translate into enhanced physical activity levels [8]. The limited effect of conventional exercise training to enhance physical activity illustrates that inducing a change in physical activity behaviour merits perhaps a more comprehensive approach than simply targeting the underlying exercise intolerance and muscle dysfunction [9].

Principles of motivational interviewing have been used to obtain lifestyle changes in several health behaviours including smoking [10], dietary habits [11], substance abuse [12] and physical activity [13]. In terms of physical activity behaviour, this patient-centred approach focuses on the identification of personal barriers precluding an increase in daily physical activity and stimulates the patients to actively search for solutions to overcome these barriers. Enhancing motivation to lead a more active life and self-efficacy to perform daily life activities plays a central role in this process.

To date, one group investigated the feasibility of a physical activity counselling program in addition to pulmonary rehabilitation in patients with COPD [14, 15]]. The authors concluded that this strategy can be effective, but changes in physical activity were rather limited (less than 20% increase) and similar to previously observed changes after pulmonary rehabilitation alone [8]. Furthermore, physical activity was measured with pedometers, which precludes investigating changes in intensity of performed physical activities. Therefore, the aim of this trial is to investigate whether the addition of a physical activity counselling program to a conventional multidisciplinary pulmonary rehabilitation program results in enhanced daily levels of physical activity as measured with validated activity monitors. The baseline and three month data obtained by the Sensewear activity monitor in this trial have been reported for the full cohort in a methodological paper by Demeyer et al [16].

## Methods

### Study design

This study is a two-armed randomized controlled trial, reported according to the 2010 CONSORT statement.

Both groups attended a comprehensive outpatient pulmonary rehabilitation program in University Hospital Gasthuisberg, Leuven. The intervention group was offered an additional physical activity counselling program, whereas the control group received equal face to face attention (sham program).

Outcome measurements were performed before and after three and six months of rehabilitation. Daily physical activity levels were assessed for seven consecutive days using two activity monitors (MoveMonitor and Sensewear Pro Armband). Clinical evaluations including assessment of pulmonary function, peripheral muscle force, six-minute walking distance and quality of life were performed at the same time points, but not on the same days. All tests were performed by experienced health professionals that were blinded to group allocation. The multidisciplinary team providing pulmonary rehabilitation was also blinded to group allocation.

Patients were informed about the study protocol prior to the start of rehabilitation. Written informed consent was obtained at that moment. Patients who agreed to participate wore the activity monitors for one week prior to start of rehabilitation and were then, after stratification for daily number of steps (< 5000 daily steps versus ≥ 5000 daily steps), randomised 1:1 into an intervention and a control group. Group allocation was performed using sealed opaque envelopes in random block sizes of four and six (unknown by the investigators)[17].

The study **was** approved by the local ethics committee (Commissie Medische Ethiek UZ Leuven) on April 15^th^ 2009 and inclusion was initialized immediately after. The study was registered at the clinicaltrials.gov online database (NCT00948623). Registration was erroneously done after commencement of inclusion, but before the first patients reached the end of the study, ensuring a prospective power calculation and choice of primary endpoint. The authors confirm that all ongoing and related trials for this intervention are registered.

### Patients

Consecutive patients with stable COPD that were referred for outpatient pulmonary were screened for inclusion between April 2009 and August 2011. Follow-up was finished in February 2012. Exclusion criteria were diagnoses other than COPD, inability to walk without walking aids, orthopaedic problems impairing daily activities, diagnosed psychiatric or cognitive disorders, progressive neurological or neuromuscular disorders, nickel allergy (which precludes measurement of physical activity with the Sensewear device) and a hospitalisation during the previous four weeks. Patients who did not speak the Dutch language were also excluded.

### Pulmonary rehabilitation

Patients in both groups attended a six-month comprehensive multidisciplinary pulmonary rehabilitation program including exercise training as a key component. Individual appointments with other health care providers (pulmonologist, psychologist, occupational therapist, dietician, social worker, respiratory nurse) were scheduled and repeated if deemed necessary. These professionals were unaware of the study. An education program included sessions about understanding their disease, the role of exercise training, dealing with breathlessness, adequate inhaler use, advice on how to adapt daily life activities, psychological aspects, nutritional aspects and social and financial aspects. Each session was provided by a member of the multidisciplinary team. No specific education session on changes in daily physical activity was included, although all patients were informed that being more active in daily life is important to obtain long-term health benefits. During exercise training, patients performed cycling exercise, treadmill walking, stair climbing, arm ergometry and resistance training of upper and lower limbs [18]. Training frequency was three sessions per week during the first three months and two sessions per week during the second three month period. Training duration increased from 40–60 minutes at the start of the program to 60–90 minutes after 6 months, including resting periods. Patients performed endurance training or interval training at moderate to high intensity (initially 60% to 70% of maximal workload achieved during maximal incremental cycle ergometry and mean speed as measured during a six-minute walking test). Resistance training consisted of three sets of eight repetitions with an intensity of 70% of one-repetition maximum. The overall training load was increased gradually during the course of the program, using a Borg scale rating of 4 to 6 on dyspnoea or perceived exertion as an indicator of adequate training intensity [19].

### Physical activity counselling program and sham attention program

The intervention group participated in a physical activity counselling program, consisting of eight individual sessions. These sessions were spread out over the six-month rehabilitation program. Each session lasted for 20 to 30 minutes. During the initial counselling session, the patients’ motivation to change physical activity behaviour and their confidence to actually perform this change (self-efficacy) were evaluated on a scale from 0 to 10 [20, 21]. Physical activity levels were assessed objectively during the week prior to each session, using a Sensewear Pro Armband. During the counselling sessions, feedback on physical activity levels was provided and, depending on the patient’s motivation to change, different counselling approaches were used. The communication style used during the counselling sessions emphasized a collaborative, empathic, non-judgmental atmosphere. When patients reported high scores on motivation to change (≥8/10) the focus of the counselling intervention was on action planning, goal setting, facilitating barrier identification, and relapse prevention [22]. In the case of low scores on motivation (≤8/10) we aimed to enhance motivation by using a communicative approach based on motivational interviewing. We tried to help these patients to explore their ambivalence towards change and to express their reasons for change (e.g. a decisional balance exercise was performed by asking individuals to list benefits and costs of changing and not changing behavior in parallel columns). In contrast to an expert model (asking questions and giving advice) we followed the spirit of motivational interviewing to gradually enhance motivation of these patients towards behaviour change. All counselling sessions were carried out before or after the regular exercise training sessions by a research assistant that was briefly trained in the principles of motivational interviewing prior to start of the study. All research assistants were Master in Science in Physiotherapy and had no prior experience with the motivational interviewing approach. They all had however an exercise physiology background and specific expertise in respiratory physiotherapy. Prior to the start of inclusion, three individual training sessions of 60 minutes in the principles of motivational interviewing were organised by an experienced health psychologist (FD). No formal assessment was performed to grade the skill levels of the providers, but interviews with patients were video-taped and discussed with the health psychologist involved in the study design. The latter was done in individual or group sessions over the first few months of the study.

Patients in the control group received a sham attention program. Duration and timing of the individualized sessions were similar to the intervention group, but the general health status of the patient and the progression during training was discussed during the conversations. Intermediate evaluation of physical activity was performed, but no structured feedback was provided.

### Physical activity assessments

Physical activity measurements were performed with two activity monitors, the Minimod^®^ (McRoberts BV, The Hague, the Netherlands) and the SenseWear Pro Armband (SWA; BodyMedia, Inc., Pittsburgh, PA, USA).

The Minimod is a small (64x62x13mm) and lightweight device (68gram, including batteries) that contains a three-axial piezocapacitive sensor measuring at high time-resolution (100Hz). The piezocapacitive sensor enables the measurement of both static and dynamic accelerations in a range of -2/+2g with a resolution of 2mg. In this trial the device was used to assess daily walking time.The Minimod was inserted in an elastic belt and positioned on the lower back at the height of the second lumbar vertebra, nearby the body’s centre of mass, according to the instructions of the manufacturer. This device has recently been validated in patients with COPD [23].

The Sensewear Pro Armband (85x54x20mm, 79g) is worn on the right upper arm and integrates information from a biaxial accelerometer with signals from non-invasive sensors measuring physical parameters such as changes in body temperature, near body ambient temperature, heat flux, and galvanic skin resistance. Together with individual characteristics including gender, age, height and body mass these monitors’ outcomes are used to estimate energy expenditure utilizing proprietary equations developed by the manufacturer. The device has recently been validated in patients with COPD [24, 25]. After analysis of raw data the daily time spent performing various intensities of activity (expressed in metabolic equivalents or METs) were evaluated. In this trial, the time spent at an energy expenditure above 3.6 METs was considered at least moderate intense activity time. The time spent at an energy expenditure above 2.0 METs was considered at least mild intense activity time.

Daily walking time (MoveMonitor) and daily time spent in at least moderate intense activities (the time spent at an energy expenditure above 3.6 metabolic equivalents of task (METs); SenseWear) were a priori defined as primary outcomes of the study. Daily steps (MoveMonitor) and time spent in at least mild intense activities (the time spent at an energy expenditure above 2.0 METs; SenseWear) were secondary outcomes.

Assessments were performed on 7 consecutive days and patients were instructed to wear the activity monitors continuously from waking up until going to bed, except during showering or bathing. A break of one week was introduced in the rehab program to perform the three months evaluation. Days during which patients wore an activity monitor for less than 8 hours were not counted when calculating average values. Valid measurements were obtained on 5±1 days (baseline), 5±1 days (three months) and 4±1 days (six months) respectively. Only week days (Monday to Friday) were used in the analysis to minimise the measurement variability [16].

### Clinical assessments

Spirometry and whole body plethysmography were performed according to the European Respiratory Society guidelines for pulmonary function testing (Jaeger Master Screen Body; CareFusion; Germany)[26]. Diffusing capacity for carbon monoxide was measured by the single breath method [27].

Functional exercise performance was measured by a 6MWD test in a 50m corridor. Standardized encouragement was provided [28]. The best of two tests was used and related to reference values [29].

Isometric quadriceps force was quantified using a Cybex Norm Dynamometer (Cybex^®^ Norm, Enraf Nonius, Delft, the Netherlands). Peak extension torque was measured at 60° of knee flexion. At least 3 measurements were obtained and the highest reproducible value was taken into analysis. Reference values have been developed in our laboratory [30].

The Chronic Respiratory Disease Questionnaire (CRDQ) was used to assess health-related quality of life [31]. This 20-item questionnaire scores quality of life in 4 domains (dyspnoea, mastery, emotional functioning and fatigue). Higher scores indicate a better quality of life.

### Statistical analysis

We anticipated a mean increase in daily walking time of 10 ± 20 minutes/day after 6 months of rehabilitation in the control group (based on findings of Pitta et al [32]) and of 25 ± 20 minutes/day in the intervention group. The additional 15 minutes increase were based on average improvements of ~2000 steps (equivalent to about 20 minutes of daily walking) or ~30% increase in physical activity that were reported after pedometer based physical activity counselling interventions in various populations [33]. Twenty-nine patients in every group were needed to show a statistically significant difference between groups with a degree of certainty (statistical power) of 80% and a risk for a type I error (α) < 5%. Anticipating a drop-out rate of about 40%, based on typical drop-out numbers in our six-month program [32], a total number of 80 patients were randomised.

All statistical analyses were performed with SAS 9.3. Continuous variables were expressed as means ± standard deviations or as medians [interquartile range; IQR] (if data were not normally distributed). All statistical analyses were performed with SAS 9.3. Continuous variables were expressed as means ± standard deviations or as medians [interquartile range; IQR] (if data were not normally distributed). Intervention*time interaction effects were evaluated using mixed models (after log transformation of physical activity variables which were not normally distributed). Daylight time (as a proxy for season) was systematically included as a covariate in the model. Time between sunrise and sunset was 763±198 min at baseline, 733±169 min at three months and 704±181 min at six months. Post-hoc comparisons were performed with Tukey tests. Baseline physical (in)activity (based on median split of 33 minutes of walking per day) was added to the model to compare changes in physical activity between inactive and active patients.

We only performed a per-protocol analysis. Intention-to-treat analysis was not possible as we did not obtain follow-up data in patients that dropped out from the study.

## Results

**Fig 1:**
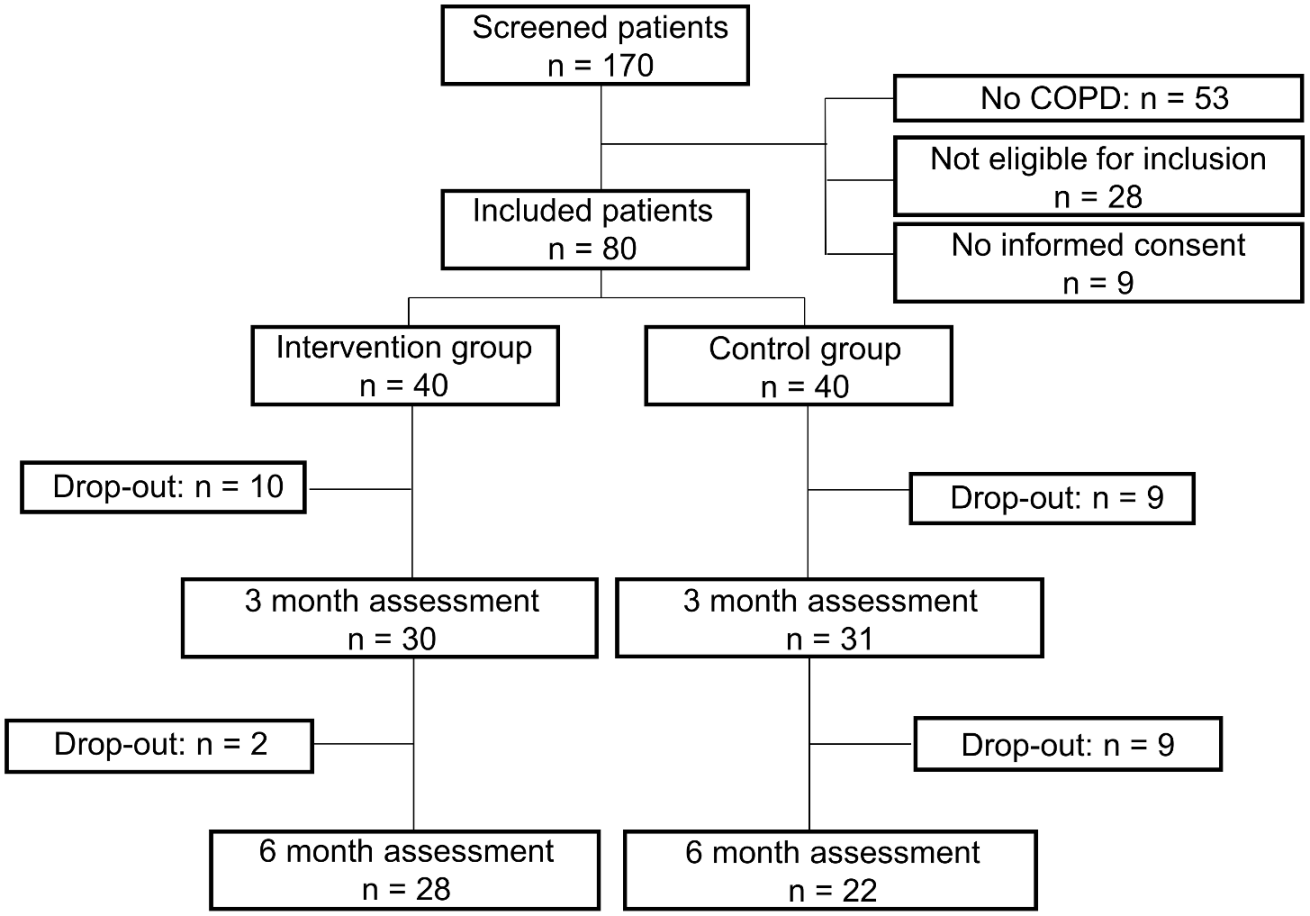
Patient flow chart.

The patient flow is summarised in Fig 1. One hundred and seventy patients were screened before the start of rehabilitation and 89 were eligible for inclusion. Main reasons for ineligibility were the absence of COPD, nickel allergy, orthopaedic problems influencing daily physical activity levels, inability to speak Dutch and cognitive disorders. Nine patients declined to participate, hence 80 patients (age 66±7 yrs, 81% male, FEV_1_ 45±16% of the predicted value) were randomised into the intervention (n = 40) and the control group (n = 40). During the first three months of rehabilitation, 19 patients (23%) dropped out of the study (10 in the intervention group, 9 in the control group), whereas 11 (14%) patients dropped out during the second three-month period (2 in the intervention group, 9 in the control group). Consequently, 28 patients in the intervention group and 22 patients in the control group completed the intervention (62.5% of the initial study population). Eighty-two per cent of planned counselling sessions were actually delivered to the patients who completed the study. Two patients withdrew their consent during the program, whereas the remained dropped out of the rehabilitation program, due to severe respiratory worsening (n = 6), orthopaedic problems (n = 3), oncological problems (n = 3), or personal reasons (n = 16) and were either not capable or unwilling to perform any follow up measurements and therefore lost-to follow up.

Baseline characteristics (Table 1) are similar in the intervention and control groups with the exception of measurements of daily physical activity, with median values that were higher in the intervention group. All baseline measurements (including measurements of physical activity) were comparable in patients who completed the study and those who dropped out. Results at three months were similar when including or excluding those patients that dropped out from the study in the second part of the six-month program. Therefore only results of patients that completed the six-month program are shown.

**Table 1:** Baseline characteristics.

Variable	Intervention (n = 40)	Control (n = 40)
Age (yrs)	66±7	67±8
Gender (% male)	86	79
BMI (kgm^-2^)	26±6	25±6
FEV_1_ (%pred)	45±14	46±18
FEV_1_/FVC (%)	40±10	41±12
FRC (%pred)	156±35	159±41
TLco (%pred)	45±14	52±19
6MWD (m)	418±103	420±115
6MWD (%pred)	64±16	66±18
QF (%pred)	69±17	77±22
CRDQ total score (20–100)	78±17	84±13
Daily walking time (min)	33 [16–47]	29 [17–44]
Daily steps (n)	3408 [1732–5709]	2574 [1592–4631]
Daily Time > 3.6 METs (min)	17 [4–50]	12 [2–26]
Daily Time > 2.0 METs (min)	40 [17–109]	34 [17–69]

Data are mean ± std or median [interquartile range]. BMI = body mass index, FEV1 = forced expiratory volume in one second, FRC = functional residual capacity, TLco = diffusion capacity for carbon monoxide, 6MWD = six-minute walking distance, QF = quadriceps force, CRDQ = Chronic Respiratory Disease Questionnaire, Daily time > 3.6 METs = daily time spent in activities with an intensity of at least 3.6 metabolic equivalents (moderate and vigorously intense activity), Daily time > 2.0 METs = daily time spent in activities with an intensity of at least 2.0 metabolic equivalents (mild, moderate and vigorously intense activity).

**Fig 2:**
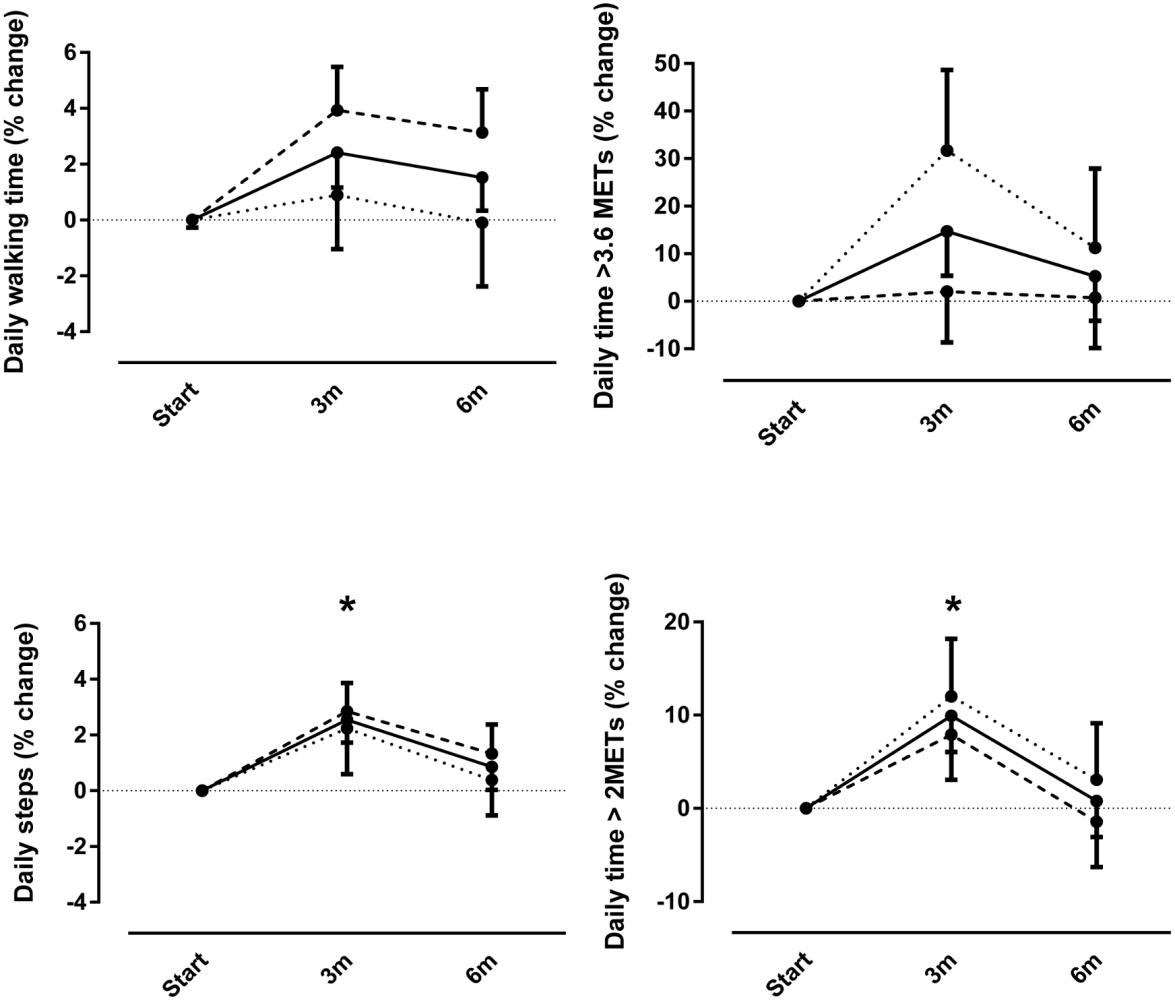
Relative changes in daily time spent walking, daily steps, daily time spent in at least moderate intense activities (>3.6 metabolic equivalents) and at least mild intense activities (>2.0 metabolic equivalents) after three months (3m) and after six months of rehabilitation (6m) compared to baseline. Data are expressed as percentage of change of least square means compared to baseline. No intervention*time effects were observed. * indicates time effect for the whole group (p<0.05 compared to baseline).

Fig 2 depicts the relative changes in physical activity levels after three and six months of rehabilitation compared to baseline. No intervention*time interaction effects were observed for the physical activity outcomes. A significant time effect (p<0.05) for the whole group was observed for daily steps and daily time spent in at least mild intense (>2.0 METs) activities but not for daily walking time and daily time spent in at least moderate intense (>3.6 METs) activities. At three months, steps (+3%) and daily time spent in at least mild intense activity (+10%) were significantly higher compared to baseline, but not at six months (+1% in both variables).

**Fig 3:**
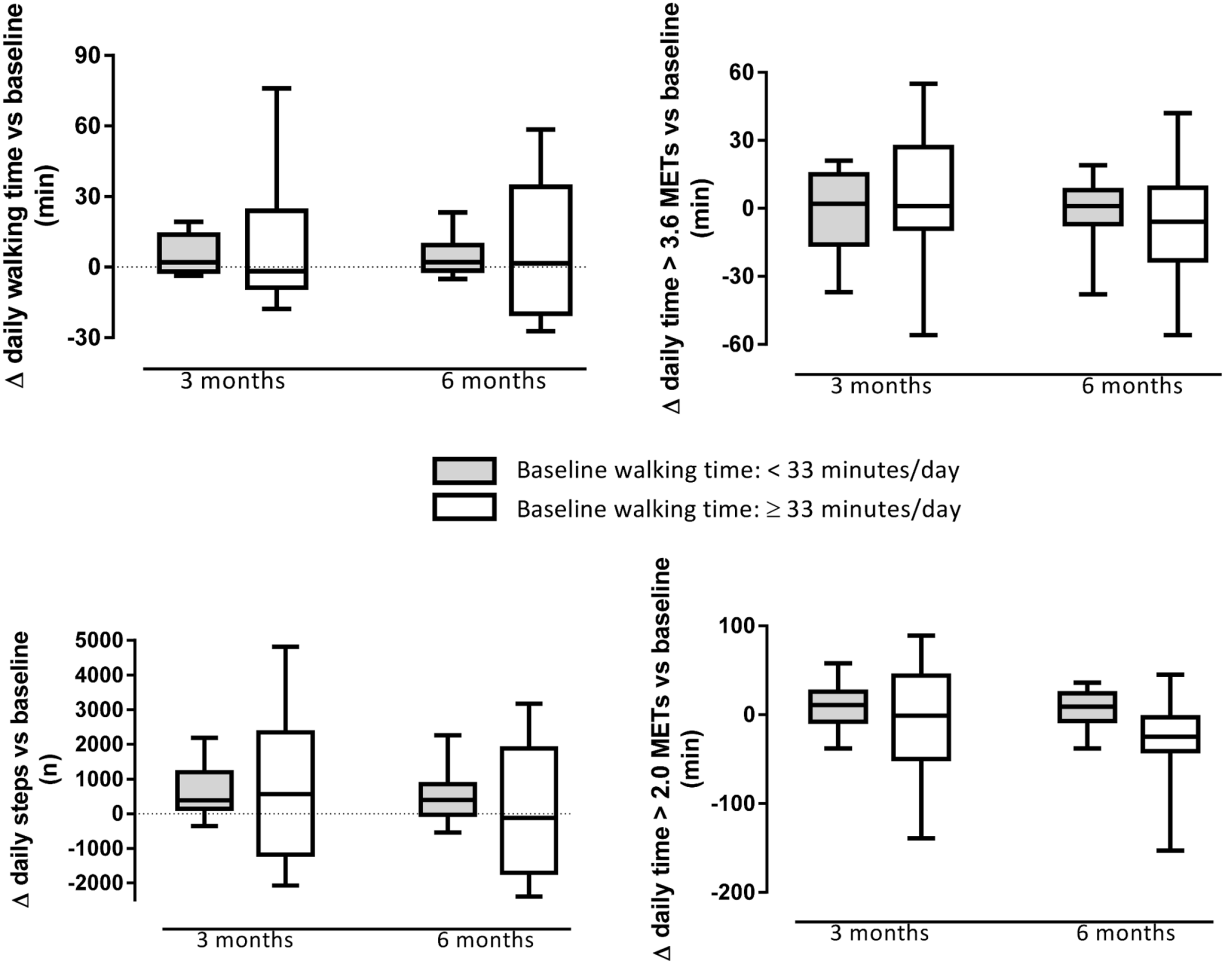
Absolute changes in daily time spent walking, daily steps, daily time spent in at least moderate intense activities (>3.6 metabolic equivalents) and at least mild intense activities (>2.0 metabolic equivalents) after three months (3m) and after six months of rehabilitation (6m) in active and inactive patients at baseline. A median split of baseline physical activity level is performed for each variable. Data are analysed using mixed models including daylight time as covariate. Box plots indicate median, quartile 1 and 3 and percentile 10 and 90. No Interaction effects (p<0.05) between baseline physical activity and time were identified.

Fig 3 reflects changes in physical activity in patients that are inactive or active at baseline (based on a median split for daily walking time). No interaction effects between baseline physical activity and intervention effects were observed (all p-values > 0.05). Physical activity did not change differently in inactive and active patients at baseline (all p-values > 0.05). Fig 3 suggests however that active patients have a more heterogeneous response in terms of physical activity.

Patients in the intervention and control group obtained similar increases in six minute walking distance (47±78 vs. 36±51m at 3 months; 43±101 vs. 36±58m at 6 months vs. baseline), quadriceps force (14±26 vs. 12±24% at 3 months; 13±23 vs. 9±14% at 6 months vs. baseline), and CRDQ score (23±14 vs. 19±12 points at 3 months; 19±21 vs. 17±14 points at 6 months vs. baseline). Results after three and six months of rehabilitation were significantly higher compared to baseline values. Lung function measurements did not change throughout rehabilitation in either study group.

## Discussion

This study aimed to investigate the effectiveness of an individual activity counselling intervention in addition to pulmonary rehabilitation in terms of daily physical activity patients with moderate to very severe COPD. Despite clinically relevant improvements in exercise tolerance and health-related quality of life, improvements in daily physical activity levels were small in both groups. The addition of individual activity counselling did not enhance activity behaviour in our patient sample.

### Interpretation of findings

Our pulmonary rehabilitation program enhanced exercise tolerance (+45m in six-minute walking distance) and health-related quality of life (+21 points in CRDQ total score) in patients with moderate to very severe COPD to a similar extent as reported previously in our program [18, 32]. The observed increases in six-minute walking distance and Chronic Respiratory Disease Questionnaire score exceeded the proposed minimal important difference for these measures [34, 35]. The effects of pulmonary rehabilitation programs in terms of physical activity levels in literature are heterogeneous, ranging from no change to a 40% increase [36]. Part of the problem with all of these former trials is the absence of treatment fidelity measures. However, our findings for different physical activity outcomes lie within the range reported in literature.

Elements of motivational interviewing have been used to alter physical activity behaviour in various populations other than COPD including patients with cancer, type II diabetes [37], obesity [38], congestive heart failure [39], adults at risk for cardiovascular disease [40, 41] and healthy adults [42, 43]. Meta-analysis reveals that this intervention only yields a significant increase in physical activity levels in a minority of published trials in chronic disease populations [44].

In most studies, unfortunately, physical activity was quantified using questionnaires. The use of self-report instead of objective measurements might have overestimated changes in physical activity in the positive trials. Altenburg et al [15] evaluated the effect of adding individual activity counselling based on motivational interviewing to a rehabilitation program using a pedometer. The authors reported a 15–20% increase in daily steps in the intervention group, compared with a 5–10% decrease in the control patients who only received pulmonary rehabilitation, confirming our lack of change in physical activity after pulmonary rehabilitation [15]. Baseline physical activity levels were similar in the study of Altenburg et al. and our study. In contrast with our study, during which feedback on physical activity levels was only provided during scheduled study visits, the patients constantly received instant feedback from a pedometer to continuously provide feedback on their current physical activity levels [15]. This might be a useful tool in short-term goal setting, providing an incentive towards a higher daily physical activity level [45]. The long-term effect of this intervention might be limited, as the difference between groups disappeared one year after the end of the intervention in the study of Altenburg et al [15].

The minimal important difference in terms of physical activity outcomes has not yet been reported in patients with COPD. ASCM guidelines propose that elderly people should engage in 30 minutes of moderate intense physical activity on at least five days per week in order to improve and maintain their health [46]. It is suggested that this translates to a daily average step count of approximately 7000 steps [47]. However, in patients with advanced COPD these goals might not be realistic [48]. The aim of an activity enhancing intervention should be to reduce sedentary behaviour and to set individual goals of physical activity in light of their disease status, symptoms and past health behaviour [49].

### Methodological considerations

Our patients were markedly inactive during baseline assessment. Baseline physical activity levels were lower compared with previous studies performed in the same centre in patients with similar age and disease severity (e.g. mean daily walking time of 40 minutes compared to 44 and 55 minutes respectively)[3, 32]. We assessed physical activity behaviour with the Sensewear armband (79g) and the MoveMonitor activity monitor (68g), whereas the older trials used an older version of this device, the Dynaport activity monitor, which consists of a waist and a leg sensor, connected to a wire (total weight 375g). Possibly patients were more continuously aware of wearing an activity monitor when wearing the bulky device, which might have influenced physical activity behaviour.

Although the used devices have proven general validity to assess physical activity levels in patients with COPD [23, 24], we did not specifically calibrate every individual device. Therefore, occasional measurement errors cannot be excluded.

We could speculate that the timing of the counselling intervention was not optimal. We were targeting very inactive patients that were by default confronted with a sudden increase in the amount and intensity of their physical activities (i.e. three 90-minute high-intensity exercise sessions per week). Possibly, this might have affected the patients’ readiness for inducing an extra increase in activities at home. In a future trial, it would be interesting to provide the same intervention in the final stage of pulmonary rehabilitation (e.g. during the final four weeks) and during the first months after the end the program. This period would typically be the period that people are expected to translate the gains in physical capacity into a more active lifestyle, in order to maintain training results on the longer term. Further research is needed to investigate the mode of delivery of behavioural interventions in patients with COPD during pulmonary rehabilitation programs.

Also, we did not systematically record adherence to the pulmonary rehabilitation program. Consequently we cannot exclude the possibility that attendance rates were different between study groups. The similar increase in exercise tolerance and health-related quality of life however suggests that adherence was sufficiently high in both groups.

Lastly, we acknowledge that the health care providers did not have prior experience with the MI approach which might have been a problem. The current approach was however chosen on purpose to make our intervention clinically feasible and broadly applicable. The potential feasibility of this approach is also supported by recently published data showing that an MI intervention can be effectively applied by a physical activity specialist after two training sessions [50]. However, it would be reasonable to speculate that aspects such as training and experience of motivational interviewing methods influence the effectiveness and we cannot rule out that this might have improved the effectiveness of our intervention. Additionally both the intervention and sham control intervention were delivered by the same research assistants, but both type of sessions were recorded and viewed by a health psychologist to prevent contamination.

### Conclusions

This trial found no additional effect of eight individual activity counselling sessions during pulmonary rehabilitation to enhance physical activity levels in inactive patients with severe COPD. The optimisation of individual behavioural changes during pulmonary rehabilitation warrants further investigation.

## Supporting Information

S1 CONSORT Checklist CONSORT 2010 checklist. (DOC)

S1 Protocol Study protocol as approved by the local ethical committee. (DOC)
