# Correction: Physical Activity Counselling during Pulmonary Rehabilitation in Patients with COPD: A Randomised Controlled Trial

**DOI:** 10.1371/journal.pone.0148705

**Published:** 2016-02-01

**Authors:** Chris Burtin, Daniel Langer, Hans van Remoortel, Heleen Demeyer, Rik Gosselink, Marc Decramer, Fabienne Dobbels, Wim Janssens, Thierry Troosters

Fig 2 appears incorrectly in the published article. Please see the corrected Fig 2 here.

**Fig 2:**
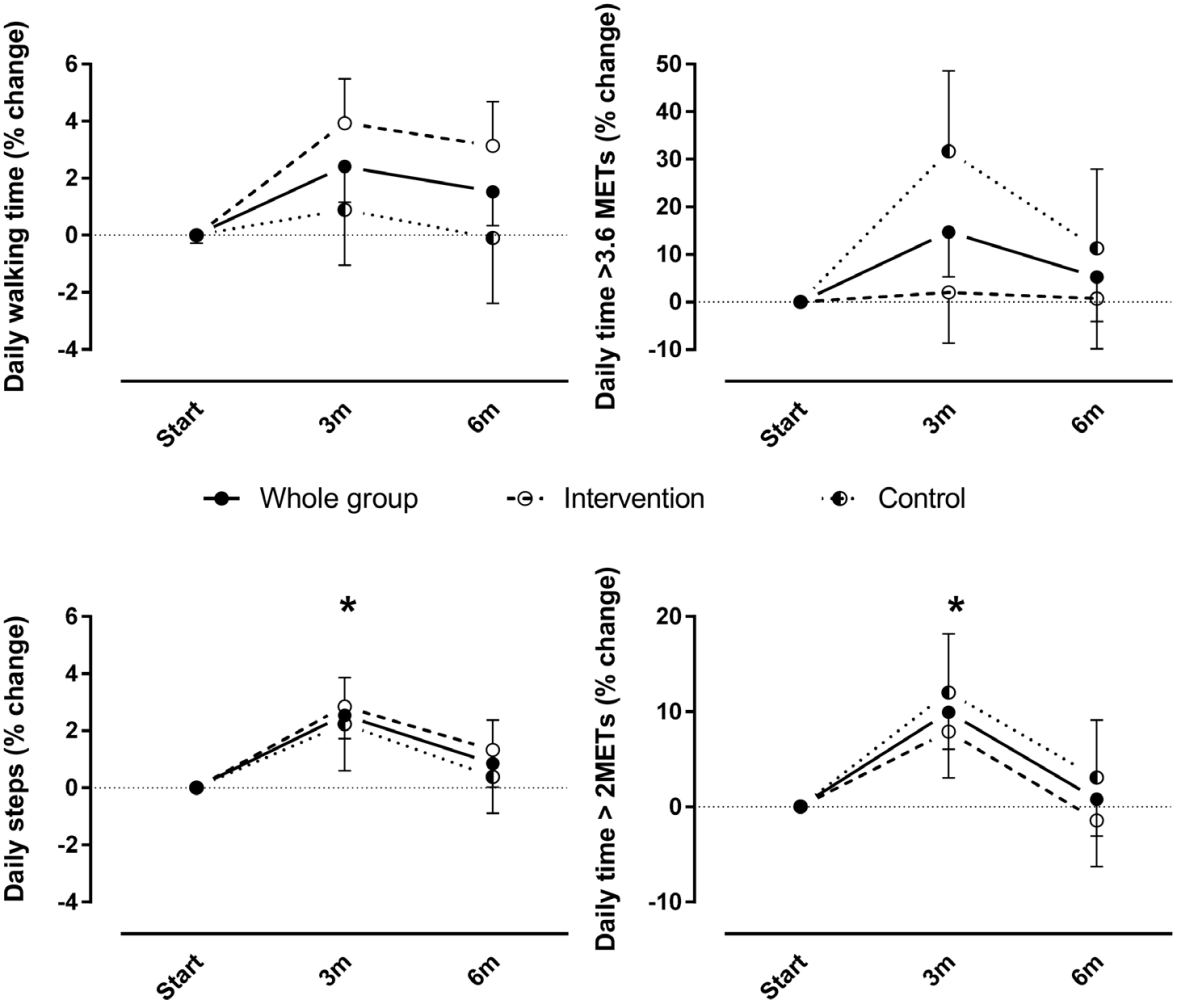
Relative changes in daily time spent walking, daily steps, daily time spent in at least moderate intense activities (>3.6 metabolic equivalents) and at least mild intense activities (>2.0 metabolic equivalents) after three months (3m) and after six months of rehabilitation (6m) compared to baseline. Data are expressed as percentage of change of least square means compared to baseline. No intervention*time effects were observed. * indicates time effect for the whole group (p<0.05 compared to baseline).
