# Supplementary material for: Physical Activity Counselling during Pulmonary Rehabilitation in Patients with COPD: A Randomised Controlled Trial
Source: PLoS One. 2015 Dec 23;10(12):e0144989. doi: 10.1371/journal.pone.0144989 (PMC4689370; doi:10.1371/journal.pone.0144989)
Supplement: S1 Protocol — (DOC) [file pone.0144989.s002.doc]

**Study protocol**

**Physical Activity Counseling during pulmonary rehabilitation:**

**Does it improve daily physical activity levels?**

**The PAC-trial**

Investigators: Chris Burtin, Daniel Langer, Hans Van Remoortel, Wim Janssens, Marc Decramer, Rik Gosselink, Thierry Troosters

Table of contents

**Introduction** [**3**](#__RefHeading___Toc216507738)

Hypothesis [4](#__RefHeading___Toc216507739)

Methods [4](#__RefHeading___Toc216507740)

Patients [4](#__RefHeading___Toc216507741)

Study design [5](#__RefHeading___Toc216507742)

Interventions [6](#__RefHeading___Toc216507743)

Pulmonary rehabilitation [6](#__RefHeading___Toc216507744)

Physical activity counseling program [6](#__RefHeading___Toc216507745)

Outcome measures [9](#__RefHeading___Toc216507746)

Statistical analysis [13](#__RefHeading___Toc216507747)

Reference List [15](#__RefHeading___Toc216507748)

# Introduction

Patients with chronic obstructive pulmonary disease (COPD) are markedly inactive in daily life^1^. Daily activity is strongly related to functional exercise capacity^1^ and is a predictor of hospital readmission^2, 3^ and mortality^3, 4^. Despite the clear beneficial effects on exercise capacity and quality of life^5^, comprehensive pulmonary rehabilitation only leads to modest improvements in daily physical activity levels^6-10^. Patients stay far from the recommended daily activity levels^11^ and the activity levels observed in healthy elderly^1^. It is unknown whether the addition of an individual counseling program leads to a more pronounced improvement. Inactivity is a disease-related health behavior and possibly a more individually tailored intervention is required to generate long-term behavioral changes. There are indications that activity counseling programs, based on cognitive behavioral strategies, could be effective in inducing long-term lifestyle changes^12, 13^. Feedback on physical activities in daily life can be provided using an activity monitor^14^. By monitoring their behavior patients will get aware of their level of daily physical activity. Based on this self-assessment, they will be able to set specific goals for increasing their physical activity. The use of activity monitors as feedback tools leads to significant increases in the level of physical activity in patients with chronic diseases and healthy subjects^15^.

The present randomized controlled trial will assess the effect of adding intensive physical activity counseling to an outpatient pulmonary rehabilitation program. Counseling including real-time feedback on daily activities from an activity monitor will be offered to improve participation in physical activities in daily life during and after an outpatient pulmonary rehabilitation program.

# Hypothesis

The addition of individually tailored activity counseling sessions, including feedback from an activity monitor, to a comprehensive pulmonary rehabilitation program improves the daily physical activity levels in patients with severe COPD. Daily number of steps and amount of time spent in moderate intense activities after 3 months of rehabilitation are considered as primary outcomes. Exercise capacity, muscle force and health-related quality of life are secondary outcomes.

# Methods

## Patients

All patients with spirometry-proven stable COPD that are referred for outpatient pulmonary rehabilitation in University Hospital Gasthuisberg, Leuven will be screened for inclusion.

Exclusion criteria:

- Diagnosed psychiatric or cognitive disorders
- Progressive neurological or neuromuscular disorders
- Orthopedic problems impairing daily activities or unable to walk without walking aids
- Patients on the waiting list for lung transplantation
- Hospitalization during the previous 3 months
- Walking speed during initial six-minute walking test < 2.5 km/h

## Study design

Patients will be informed about the study protocol prior to the start of rehabilitation. Informed consent will be obtained at that moment. Patients agreeing to participate will be randomized into an intervention and a control group. Group allocation will be performed using sealed opaque envelopes in random block sizes (unknown by the investigators) after stratification for daily number of steps at baseline (< 5000 daily steps versus ≥ 5000 daily steps; a ratio of 2:1 is expected based on findings of Pitta et al.^1^).

Both groups will follow a comprehensive pulmonary rehabilitation program. The intervention group will receive an additional activity counseling program, whereas the control group will receive a sham intervention, receiving equal face to face attention.

Daily physical activity will be assessed for seven consecutive days. Measurements will be done before, after 3 and 6 months of rehabilitation and 6 and 12 months after terminating the program. All tests will be performed by an experienced professional that is blinded to group allocation. Clinical evaluations including assessment of pulmonary function, peripheral muscle force, maximal and endurance exercise capacity, six-minute walking distance and quality of life will be performed at the same time points, but not on the same days.

## Interventions

### Pulmonary rehabilitation

Patients in both groups will follow a six-month comprehensive pulmonary rehabilitation program including exercise training as a key component. Individual appointments with other health care providers (psychologist, occupational therapist, dietician, social worker, respiratory nurse) will also be scheduled. During exercise training, patients will perform cycling, treadmill walking, stair climbing, arm ergometry and resistance training of both arm and leg muscles^16^. Training frequency will be three sessions per week during the first three months and two sessions per week during the second three month period, resulting in a total of 60 training sessions. Training duration will increase from 40-60 minutes at the start of the program to 60-90 minutes after 6 months. Patients will perform endurance training or interval training at moderate to high intensity (initially 60% to 70% of maximal workload). The overall training load will be increased gradually during the course of the program, using a Borg scale rating of 4 to 6 on perceived exertion as an indicator of adequate training intensity^17^.

### Physical activity counseling program

The intervention group will participate in a physical activity counseling program, consisting of 8 individual counseling sessions. These sessions will be spread out over the six-month rehabilitation program as illustrated in Figure 1. Each session will last for 20 to 30 minutes. Prior to each session, patients will wear an activity monitor that estimates energy expenditure on a minute-to-minute basis to evaluate physical activity behavior. Daily steps and time spent in activities of mild and moderate intensity will be registered. Evaluations of patients motivation to change physical activity behavior and their confidence to actually perform this change (self-efficacy) will be carried out^18, 19^. Throughout the intervention, patients will be asked to complete a diary about their physical activity behavior. Daily physical activity will be compared with current guidelines on the amount and intensity of daily physical activity required to achieve optimal health effects^11^. These guidelines recommend at least 30 minutes of moderate intense physical activity on most days.

The aim of the intervention is to increase patient’s daily physical activity. Daily steps and time spent in moderate intense activity will be used as markers for daily physical activity. Counseling strategies based on principles of motivational interviewing^20^ will be used to construct an individualized action plan, with the intention to evolve step by step towards a more active lifestyle. In this context, a video providing background about the process of activity behavior change will be shown to the patients. Barriers to adopt a physically active lifestyle will be identified and patients will be encouraged to actively search for solutions to overcome these barriers. Feedback on daily activities from an activity monitor will inform patients about the results of their attempts to increase daily physical activity over the course of the intervention. All counseling sessions will be carried out before or after the regular exercise training sessions by a research assistant skilled in the techniques of motivational interviewing. The sessions will be recorded on video (with permission of the patient) to perform a retrospective qualitative analysis of the counseling process.

Patients in the control group will receive a sham attention program. Duration and timing of the individualized sessions will be similar to the intervention group, but the general health status of the patient and the progression during training will be discussed during the conversations. Intermediate evaluation of physical activity will be performed, but no structured feedback will be provided. The sham intervention rules out a possible attention effect.

**Figure 1.** Schematic overview of study protocol

## Outcome measures

Table 1 gives an overview of the outcome measures at different time points in the study.

*Physical activity monitoring*

Measurements will be performed with an accelerometer-based activity monitor (Minimod®, McRoberts BV, The Hague, the Netherlands). The Minimod is a small (64x62x13mm) and lightweight device (68gram, including batteries) that contains a three-axial piezocapacitive sensor measuring at high time-resolution (100Hz). The piezocapacitive sensor enables the measurement of both static and dynamic accelerations in a range of -2/+2g with a resolution of 2mg. Analysis of raw data allows for classification of intensity, duration and frequency of movement. Different postures and walking are identified and energy expenditure is estimated. The Minimod will be inserted in an elastic belt and positioned on the lower back at the height of the second lumbar vertebra, nearby the body’s centre of mass, according to the instructions of the manufacturer. The Minimod has been recently validated in patients with COPD^21^. Assessments will be done on 7 consecutive days during 12 hours per day.

The SenseWear Pro Armband (SWA; BodyMedia, Inc., Pittsburgh, PA), will be used for feedback on and intermediate evaluation of physical activity and energy expenditure. The device (85x54x20mm, 85g) will be worn on the upper arm and integrates information from a biaxial accelerometer with signals from non-invasive sensors measuring physical parameters such as changes in body temperature, near body ambient temperature, heat flux, and galvanic skin resistance. Together with individual characteristics including gender, age, height and body mass these variables will be used to estimate energy expenditure utilizing proprietary equations developed by the manufacturer. The device has recently been validated in patients with COPD^22^. After analysis of raw data the number of steps as well as time spent performing various intensities of activity can be evaluated. Patients will regularly be confronted with their daily time spent in mild intensity physical activity (2.0 – 3.6 METs) and moderate intensity physical activity (>3.6 METs) and steps that are accumulated on a daily basis

*Pulmonary function*

Spirometry and whole body plethysmography will be performed according to the European Respiratory Society guidelines for pulmonary function testing (Vmax Autobox, Sensor Medics, Bilthoven, the Netherlands)^23^. Diffusing capacity for carbon monoxide will be measured by the single breath method (Sensor Medics 6200, Bilthoven, the Netherlands).

*Peripheral muscle force*

Isometric quadriceps force will be quantified using a Cybex Norm Dynamometer (Cybex® Norm, Enraf Nonius, Delft, the Netherlands). Peak extension torque will be measured at 60° of knee flexion. At least 3 measurements will be obtained and the highest reproducible value will be taken into analysis. Reference values have been developed in our laboratory^24^.

Isometric hand grip force will be measured using a hydraulic hand grip dynamometer (Jamar Preston, Jackson, MI). Peak force will be assessed with the elbow fixed to the rib cage and flexed 90° and with the wrist in neutral position. At least 3 measurements will be obtained and the highest reproducible value will be taken into analysis and related to reference values^25^.

*Maximal exercise capacity*

Maximal exercise capacity will be assessed by a maximal incremental cycle exercise test (Ergometrics 900, Ergoline, Bitz, Germany). After a 2-min resting period and 3 minutes of unloaded cycling, patients will start at 20 W and cycle until symptom limitation at an incremental workload of +10 W/min. Oxygen consumption, carbon dioxide output and ventilation will be measured breath by breath (Vmax series, SensorMedics, Anaheim, CA). Heart rate and oxygen saturation will be registered continuously. Maximal oxygen consumption will be compared with normal values^26^.

*Endurance exercise capacity*

A constant work rate cycle test until exhaustion will be performed at 75% of the maximal power output (in Watts) that was reached during the initial incremental exercise test (Ergometrics 900, Ergoline, Bitz, Germany). Oxygen consumption, carbon dioxide output and ventilation will be measured breath by breath (Vmax series, SensorMedics, Anaheim, CA). Heart rate and oxygen saturation will be monitored continuously. The perception of dyspnea and leg fatigue will be quantified at 2-minute intervals during exercise using the modified Borg scale^17^.

*6-minute walking distance (6MWD)*

Functional exercise performance will be measured by a six-minute walking test in a 50m corridor. Standardized encouragement will be provided^27^. The best of two tests will be used and related to reference values^28^.

*Modified Pulmonary Functional Status and Dyspnea Questionnaire (PFSDQ)*

The modified PFSDQ is a disease specific self-administered questionnaire that assesses subjective aspects of activities of daily living. It consists of three components: activity, dyspnea and fatigue. For 10 activities, the level of change in performing these activities compared to the period before respiratory problems developed is reported. For the dyspnea and fatigue subscales, the severity of symptoms associated with the same 10 activities is assessed and additionally 5 general questions are asked. They assess the patient’s experience with dyspnea/fatigue, the frequency of occurrence during the past month and the intensity of these symptoms on most days, today and during usual activities respectively. All items are rated on a 0 to 10 ordinal scale. For each of the three components, lower scores indicate a better status.

*Health-related quality of life*

The Chronic Respiratory Disease Questionnaire (CRDQ) will be used to assess health-related quality of life^29^. This 20-item questionnaire scores quality of life in 4 domains (dyspnea, mastery, emotional functioning and fatigue) and has been validated in the Dutch language^30^.The total score can range from 20 to 140 with higher scores indicating better quality of life.

*Self efficacy for walking*

The used scale to assess self efficacy for walking has been adapted from Kaplan et al^31^. The scale includes brief statements describing progressively more difficult performance requirements for walking. It consists of the following statements: how sure are you that you can walk 300m (about 5 min), walk 600m (about 10 min), walk 900m (about 15 min),…,walk 5400m (about 90 min). For each item, the patient rates the degree of confidence to perform that activity on a 10- point probability scale, ranging in 10-point intervals from *complete uncertainty* (0) to *complete certainty* (10). The score reflects the highest level that the patient expressed 100% confidence he or she could perform. If the highest level for which a patient indicated 100% confidence was the lowest level (walk 300m), a score of 1 was assigned. If the highest level of 100% confidence was the third level (walk 900m), a score of 3 was assigned, and so on.

*Anxiety and depression*

The Hospital Anxiety and Depression Scale (HADS) will be used to assess emotional distress. The HADS consists of 14 items and has separate scores for anxiety (7 items) and depression (7 items). A score of 11 or greater on either of the sub-scales suggests clinically significant symptoms of anxiety or depression.

**Table 1. Overview of outcome measurements.**

|  | Start | 3 months | 6 months | 6 months post | 12 months post |
| --- | --- | --- | --- | --- | --- |
| Screen | X |  |  |  |  |
| Informed Consent | X |  |  |  |  |
| Daily physical activity (7 days):   - Minimod - Armband | X  X | X  X | X  X | X  X | X  X |
| Pulmonary function | X | X | X | X | X |
| Quadriceps force | X | X | X | X | X |
| Handgrip force | X | X | X | X | X |
| Maximal exercise capacity | X | X | X | X | X |
| Endurance exercise capacity | X | X | X | X | X |
| Six-minute walking distance | X | X | X | X | X |
| PFSDQ | X | X | X | X | X |
| CRDQ | X | X | X | X | X |
| Self-efficacy for walking | X | X | X | X | X |
| HADS | X | X | X | X | X |

## Statistical analysis

Walking time in daily life and the amount of time spent in moderate intense activities will be considered as primary outcomes.

We anticipate a mean increase in daily walking time of 10 ± 20 minutes/day after 6 months of rehabilitation in the control group (based on findings of Pitta et al.^6^) and of 25 ± 20 minutes/day in the intervention group. Twenty-nine patients in every group will be needed to show a statistically significant difference between groups with a degree of certainty (statistical power) of 80% and a risk for a type I error (α) < 5%. Anticipating a drop-out rate of 40%, a total number of 80 patients will be randomized.

Continuous variables will be expressed as means ± standard deviations (if data are normally distributed) or as medians [interquartile range; IQR] (if data are not normally distributed). Differences between groups will be evaluated using unpaired t-tests or Wilcoxon-Mann Whitney tests. The within-group differences between baseline and 6 month results will be analysed using paired t-tests whereas the intervention-time effects will be evaluated using repeated-measures ANOVA. The type I error will be 0.05 for all statistical tests. If baseline imbalances will occur in potentially confounding variables, they will be corrected for using ANCOVA^32^.

# Reference List

(1) Pitta F, Troosters T, Spruit MA, Probst VS, Decramer M, Gosselink R. Characteristics of physical activities in daily life in chronic obstructive pulmonary disease. *Am J Respir Crit Care Med* 2005 May 1;171(9):972-7.

(2) Garcia-Aymerich J, Farrero E, Felez MA, Izquierdo J, Marrades RM, Anto JM. Risk factors of readmission to hospital for a COPD exacerbation: a prospective study. *Thorax* 2003 February;58(2):100-5.

(3) Garcia-Aymerich J, Lange P, Benet M, Schnohr P, Anto JM. Regular physical activity reduces hospital admission and mortality in chronic obstructive pulmonary disease: a population based cohort study. *Thorax* 2006 September;61(9):772-8.

(4) Yohannes AM, Baldwin RC, Connolly M. Mortality predictors in disabling chronic obstructive pulmonary disease in old age. *Age Ageing* 2002 March;31(2):137-40.

(5) Lacasse Y, Wong E, Guyatt GH, King D, Cook DJ, Goldstein RS. Meta-analysis of respiratory rehabilitation in chronic obstructive pulmonary disease. *Lancet* 1996 October 26;348(9035):1115-9.

(6) Pitta F, Troosters T, Probst VS, Langer D, Decramer M, Gosselink R. ARE PATIENTS WITH COPD MORE ACTIVE AFTER PULMONARY REHABILITATION ? *Chest* 2008 April 10.

(7) Coronado M, Janssens JP, de MB, Terrier P, Schutz Y, Fitting JW. Walking activity measured by accelerometry during respiratory rehabilitation. *J Cardiopulm Rehabil* 2003 September;23(5):357-64.

(8) Steele BG, Belza B, Hunziker J et al. Monitoring daily activity during pulmonary rehabilitation using a triaxial accelerometer. *J Cardiopulm Rehabil* 2003 March;23(2):139-42.

(9) Sewell L, Singh SJ, Williams JE, Collier R, Morgan MD. Can individualized rehabilitation improve functional independence in elderly patients with COPD? *Chest* 2005 September;128(3):1194-200.

(10) Mercken EM, Hageman GJ, Schols AM, Akkermans MA, Bast A, Wouters EF. Rehabilitation decreases exercise-induced oxidative stress in chronic obstructive pulmonary disease. *Am J Respir Crit Care Med* 2005 October 15;172(8):994-1001.

(11) Haskell WL, Lee IM, Pate RR et al. Physical activity and public health: updated recommendation for adults from the American College of Sports Medicine and the American Heart Association. *Circulation* 2007 August 28;116(9):1081-93.

(12) Atkins CJ, Kaplan RM, Timms RM, Reinsch S, Lofback K. Behavioral exercise programs in the management of chronic obstructive pulmonary disease. *J Consult Clin Psychol* 1984 August;52(4):591-603.

(13) de Blok BM, de Greef MH, ten Hacken NH, Sprenger SR, Postema K, Wempe JB. The effects of a lifestyle physical activity counseling program with feedback of a pedometer during pulmonary rehabilitation in patients with COPD: a pilot study. *Patient Educ Couns* 2006 April;61(1):48-55.

(14) Pitta F, Troosters T, Probst VS, Spruit MA, Decramer M, Gosselink R. Quantifying physical activity in daily life with questionnaires and motion sensors in COPD. *Eur Respir J* 2006 May;27(5):1040-55.

(15) Bravata DM, Smith-Spangler C, Sundaram V et al. Using pedometers to increase physical activity and improve health: a systematic review. *JAMA* 2007 November 21;298(19):2296-304.

(16) Troosters T, Gosselink R, Decramer M. Short- and long-term effects of outpatient rehabilitation in patients with chronic obstructive pulmonary disease: a randomized trial. *Am J Med* 2000 August 15;109(3):207-12.

(17) Borg GA. Psychophysical bases of perceived exertion. *Med Sci Sports Exerc* 1982;14(5):377-81.

(18) Miller WR. Enhancing patient motivation for health behavior change. *J Cardiopulm Rehabil* 2005 July;25(4):207-9.

(19) Kaplan RM, Ries AL, Prewitt LM, Eakin E. Self-efficacy expectations predict survival for patients with chronic obstructive pulmonary disease. *Health Psychol* 1994 July;13(4):366-8.

(20) Emmons KM, Rollnick S. Motivational interviewing in health care settings. Opportunities and limitations. *Am J Prev Med* 2001 January;20(1):68-74.

(21) Langer D. Activity monitoring in patients with COPD: validation of a device to detect postures and energy expenditure. 2008.
Ref Type: Unpublished Work

(22) Patel SA, Benzo RP, Slivka WA, Sciurba FC. Activity monitoring and energy expenditure in COPD patients: a validation study. *COPD* 2007 June;4(2):107-12.

(23) Quanjer PH, Tammeling GJ, Cotes JE, Pedersen OF, Peslin R, Yernault JC. Lung volumes and forced ventilatory flows. Report Working Party Standardization of Lung Function Tests, European Community for Steel and Coal. Official Statement of the European Respiratory Society. *Eur Respir J Suppl* 1993 March;16:5-40.

(24) Decramer M, Lacquet LM, Fagard R, Rogiers P. Corticosteroids contribute to muscle weakness in chronic airflow obstruction. *Am J Respir Crit Care Med* 1994 July;150(1):11-6.

(25) Mathiowetz V, Kashman N, Volland G, Weber K, Dowe M, Rogers S. Grip and pinch strength: normative data for adults. *Arch Phys Med Rehabil* 1985 February;66(2):69-74.

(26) Jones NL, Makrides L, Hitchcock C, Chypchar T, McCartney N. Normal standards for an incremental progressive cycle ergometer test. *Am Rev Respir Dis* 1985 May;131(5):700-8.

(27) Guyatt GH, Pugsley SO, Sullivan MJ et al. Effect of encouragement on walking test performance. *Thorax* 1984 November;39(11):818-22.

(28) Troosters T, Gosselink R, Decramer M. Six minute walking distance in healthy elderly subjects. *Eur Respir J* 1999 August;14(2):270-4.

(29) Guyatt GH, Berman LB, Townsend M, Pugsley SO, Chambers LW. A measure of quality of life for clinical trials in chronic lung disease. *Thorax* 1987 October;42(10):773-8.

(30) Gosselink HAAM, Wagenaar RC, Keimpema VA, Chadwick-Straver RVM. The effects of a rehabilitation program in patients with COPD and asthma. Ned.Tijdschr.Fysioth. 100, 193-199. 1990.
Ref Type: Abstract

(31) Kaplan RM, Atkins CJ, Reinsch S. Specific efficacy expectations mediate exercise compliance in patients with COPD. *Health Psychol* 1984;3(3):223-42.

(32) Altman DG, Schulz KF, Moher D et al. The revised CONSORT statement for reporting randomized trials: explanation and elaboration. *Ann Intern Med* 2001 April 17;134(8):663-94.
